# Nanophotonic quantum sensing with engineered spin-optic coupling

**DOI:** 10.1515/nanoph-2022-0682

**Published:** 2023-01-09

**Authors:** Laura Kim, Hyeongrak Choi, Matthew E. Trusheim, Hanfeng Wang, Dirk R. Englund

**Affiliations:** Research Laboratory of Electronics, MIT, Cambridge, MA 02139, USA; Department of Materials Science and Engineering, University of California, Los Angeles, CA 90095, USA; Department of Electrical Engineering and Computer Science, MIT, Cambridge, MA 02139, USA; U.S. Army Research Laboratory, Sensors and Electron Devices Directorate, Adelphi, MD 20783, USA

**Keywords:** IR absorption readout, magnetic imaging, magnetometry, NV diamond, quantum diamond microscopy, quantum sensing

## Abstract

Nitrogen vacancy centers in diamond provide a spin-based qubit system with long coherence time even at room temperature, making them suitable ambient-condition quantum sensors for quantities including electromagnetic fields, temperature, and rotation. The optically addressable level structures of NV spins allow transduction of spin information onto light-field intensity. The sub-optimal readout fidelity of conventional fluorescence measurement remains a significant drawback for room-temperature ensemble sensing. Here, we discuss nanophotonic interfaces that provide opportunities to achieve near-unity readout fidelity based on IR absorption via resonantly enhanced spin-optic coupling. Spin-coupled resonant nanophotonic devices are projected to particularly benefit applications that utilize micro- to nanoscale sensing volume and to outperform present methods in their volume-normalized sensitivity.

## 1 Overview

Quantum sensors based on spin qubits rely on the relative phase between two quantum states accumulated during a coherent precession time under external perturbation, typically a magnetic field. This spin precession persists until the superposition state decoheres due to environmental noises. Thus, a maximum achievable sensitivity depends on the coherence time of spin qubits. Solid-state spin systems have emerged as a leading quantum sensing platform as they mimic isolated atoms with level structures decoupled from the states of a host wide-bandgap material [1]. In particular, nitrogen-vacancy (NV) centers in diamond have shown coherence time exceeding milliseconds even at room temperature [2, 3]. NV centers now set the state-of-the-art for many regimes of quantum sensing, from bulk vector magnetometers to the detection of nuclear magnetic resonance on the scale of single molecules [4].

The spin coherence time of a few μs achievable in commercially available diamond implies a spin-projection-noise-limited sensitivity ( $\eta_{\text{sp}}=\frac{1}{\gamma_{\text{e}}\sqrt{\tau }}$ , where *γ*_e_ is the electron gyromagnetic ratio and *τ* is the sensing time) on the order of nT for one-second integration, which is comparable to the magnetic field generated by a single electron ∼50 nm away. Achieving this limit, however, requires perfect readout of the spin population. Indirectly probing spins via optical measurements introduces an additional measurement uncertainty. An imperfect readout of the spin states prevents us from reaching the spin projection limit (i.e., *η*_sp_ is degraded by a factor of the inverse of readout fidelity, *σ*_R_ = 1/*F*). 

Near-unity readout fidelity has been demonstrated with a single NV electron spin at cryogenic temperature using a spin-selective resonant optical transition [5, 6]. At room temperature, however, the optical transitions can no longer be resonantly addressed due to thermal homogeneous broadening. Thus, the spin readout becomes probabilistic, relying on the interplay between the rate constants of the transitions. With the conventional optical readout method, at room temperature, a *σ*_R_ as low as ∼10 has been demonstrated for a single NV [7, 8]. Ensemble sensing gives a better absolute sensitivity as the sensitivity scales as $\frac{1}{\sqrt{N}}\;$ , where *N* is the total number of non-interacting NV centers. However, ensemble sensing comes with additional challenges, including inhomogeneous linewidth broadening, inhomogeneity in microwave (MW) and optical power deliveries, and available MW/optical power budgets to address all NVs within a given volume. The best readout fidelity demonstrated with an NV ensemble remains near 100 times below the spin projection noise limit [9]. With standard optics to collect fluorescence, *σ*_R_ of the conventional fluorescence readout at room temperature with NV ensembles remains on the order of 1000 [10–12]. Improvements in the readout fidelity offer a straightforward path to dramatically improving sensitivity as well as enabling observations of quantum correlations via projective measurements.

Improving the sensitivity of NV magnetometry has been approached from many different angles: various methods to increase readout fidelity, diamond material engineering to create a clean magnetic environment for NV spins, and spin control techniques to extend the quantum coherence. For a comprehensive coverage of methods and techniques to optimize the sensitivity of NV quantum sensing, we refer interested readers to the following recent reviews [13, 14]. In this article, we discuss the opportunities for resonant nanophotonic design to enhance spin-optic coupling of solid-state ensemble quantum sensors and to achieve sensitivity near the spin projection noise limit.

## 2 Background: NV centers

**Figure 1:**
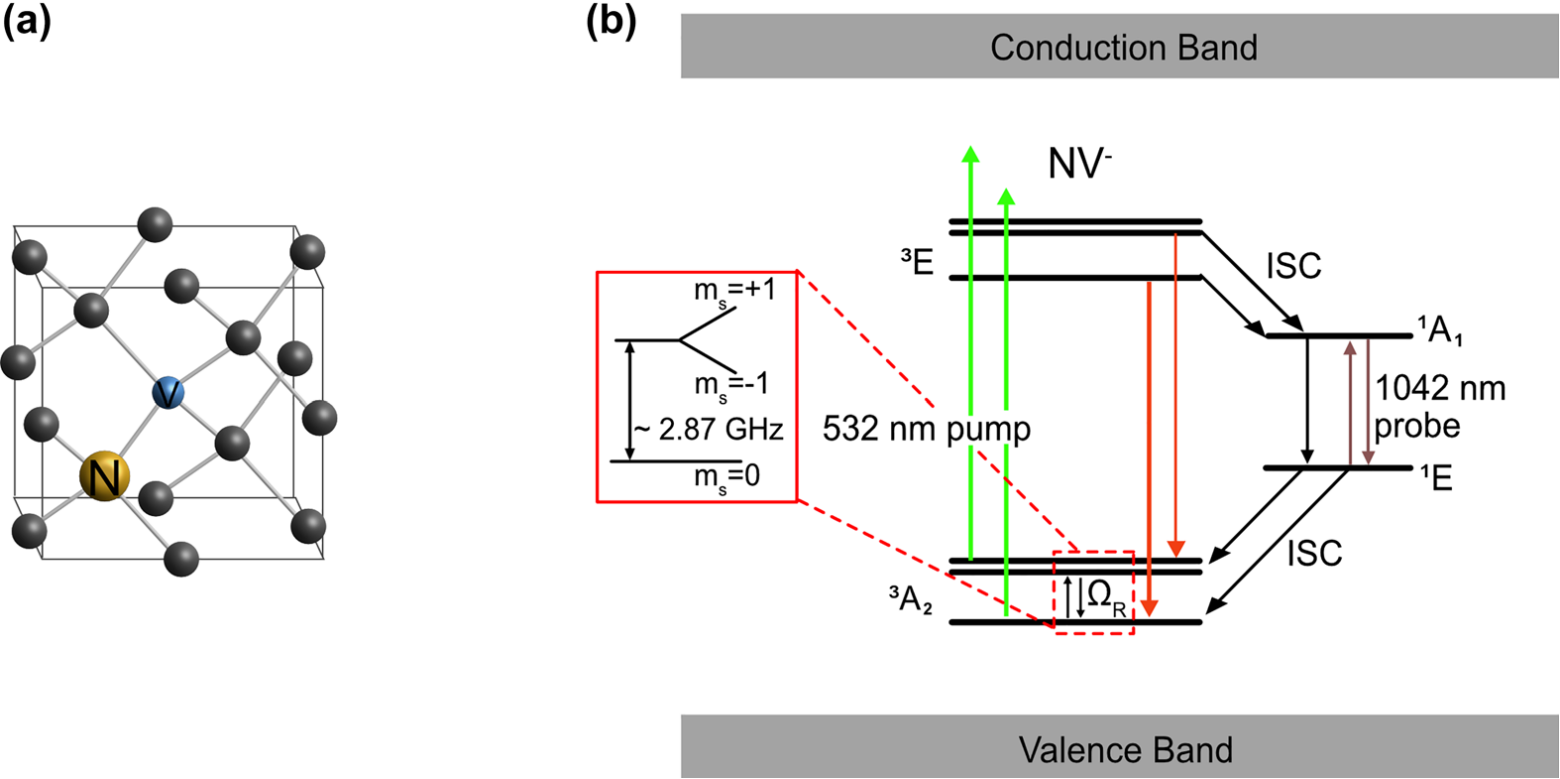
NV background: (a) NV centers in diamond crystal. The grey (yellow) sphere represents carbon (nitrogen) atoms, and the blue sphere represents a vacancy site. (b) Electronic level structure of NVs. NV^−^ has a ground spin triplet state for quantum sensing. |*m*_s_ = 0⟩ and |±1⟩ states are split by zero-field splitting (∼2.87 GHz). Because of the stronger ISC to the singlet state from |±1⟩ than |0⟩ state, |0⟩ state is brighter than |±1⟩ states under 532-nm illumination.

NV center in diamond is a substitutional nitrogen defect in a carbon lattice adjacent to a vacancy (Figure 1(a)) [15]. Negatively charged NV centers, a preferred charge state for quantum sensing, have spin-triplet ground states (|*m*_s_⟩ = |0, ±1⟩) as shown in Figure 1(b). In the absence of a magnetic field, the states |0⟩ and |±1⟩ are separated by a zero-field splitting (ZFS) of *f*_0_ ≈ 2.87 GHz. The external magnetic field component projected along the NV axis, *B*_‖_, further splits the |1⟩ and |−1⟩ by *γ*_e_*B*_‖_, where *γ*_e_ is the gyromagnetic ratio of the electronic spin of an NV. Upon spin-conserving optical excitation, excited electrons in ^3^*E* can return to the ground states either radiatively (^3^*E*→^3^*A*_2_) or non-radiatively through the intersystem crossing (ISC) mediated transitions $\left(E_{3}\to^{1}A_{1}\to^{1}E\to^{3}A_{2}\right)\;$ . The initialization and optical readout of NVs are possible due to the ISC mechanism. The non-radiative transition to the singlet states predominantly comes from the |±1⟩ states of ^3^*E* [16], followed by a preferential decay into the *m*_s_ = |0⟩  state of ^3^*A*_2_ [17, 18]. Therefore, a few cycles of optical excitation depopulate the |±1⟩ states and spin polarize NVs into the |0⟩ state of ^3^*A*_2_ during the initialization step. With MW excitation that is resonant with |0⟩ ↔ |±1⟩ of ^3^*A*_2_, the spin population transfer to the *m*_s_ = |±1⟩ states is read-out optically by observing a reduction in collected fluorescence intensity. This reduction in fluorescence intensity allows for differentiation between the spin states and forms the basis of conventional optical spin detection.

## 3 Optical readout

Following the derivation from [7], *σ*_R_ can be written as follows:

(1) $$\sigma_{\text{R}}=\sqrt{1+\frac{1}{C^{2}n_{\text{avg}}}}$$

where *C* is the measurement contrast defined by a fractional difference in the detected optical signals between NVs in *m*_s_ = |±1⟩ and *m*_s_ = |0⟩, and *n*_avg_ is the average number of photons collected per NV spin per measurement [13].

The readout fidelity of conventional fluorescence detection is limited by the intrinsic brightness contrast between the spin states and the collection rate of emitted photons. The average number of collected photons, *n*_avg_, is limited by the shelving time in the metastable state and is further reduced by the suboptimal collection efficiency. A substantial fraction of fluorescence that is radiated gets trapped inside the high-refractive-index diamond substrate (*n*_diamond_ = 2.4) by total internal reflection, or radiated with a reduced effective numeral aperture. The typical collection efficiency from a planar diamond substrate with a high NA objective remains <10% , resulting in *C*^2^*n* ≪ 1 and making measurements photon-shot-noise-limited $\left(\sigma_{\text{R}}\approx \frac{1}{C\sqrt{n_{\text{avg}}}}\right)$ . Thus, engineering far-field radiation patterns for improved photon collection has been an area of significant effort using bulk optics and sub-wavelength nanostructures [7, 9, 19], [20], [21], [22]. In these works, the photon collection efficiency is increased while the dynamics of the NV electronic transitions remains mostly unchanged.

With resonant nanophotonic structures, on the other hand, one can modify the rate of radiative emission through density-of-states engineering while simultaneously increasing collection efficiency [23–26]. However, resonantly enhancing the radiative transitions works unfavorably for room-temperature sensing applications because the increase in brightness is accompanied by the reduced spin contrast [27]. This undesirable effect is due to the fact that resonantly enhancing both radiative transitions (*m*_s_ = |0(±1)⟩ of ^3^*E* → *m*_s_ = |0(±1)⟩ of ^3^*A*_2_) dilutes the role of branching ratio of the transitions from |±1⟩ of ^3^*E* to the ground level of ^3^*A*_2_ and to the singlet state. As discussed in [27], Purcell enhancement of the visible transitions may still improve an overall SNR in a single-NV system when it is driven near its saturation, though this was not observed in experiment.

There is a significant advantage in engineering spin-optic coupling when the readout is done via absorption, rather than emission. As the singlet state is predominantly populated from *m*_s_ = |±1|⟩, the readout of the singlet-state transition (resonant with 1042 nm light) can provide the necessary spin contrast. Importantly, Purcell enhancement of the singlet-state transition does not alter the branching ratio of ^3^*E* → ^3^*A*_2_ and ^3^*E* → ^1^*A*_1_, and thus can work favorably to improve the measurement spin contrast. Because the lifetime of ^1^*A*_1_ is approximately two orders of magnitude shorter than that of ^1^*E*  [28], the singlet-state transition has an unusually high saturation intentiy that is orders of magnitude higher than that of the visible transitions. Due to this lifetime imbalance, the spin population in ^1^*E* can be read-out by observing the absorption signal at 1042 nm. An immediate advantage of this absorption method is a near-unity collection efficiency achievable with a directional IR probe beam. Moreover, this absorption-based readout is non-destructive. During the lifetime of ^1^*E*, each NV can absorb more than one photon per cycle, improving the SNR, until the system is driven near the saturation level. Lastly, one can adopt coherent detection methods, such as homodyne or heterodyne measurements, which can be especially helpful for systems with large electrical noise.

The biggest drawback in achieving high spin contrast via the absorption readout is its small absorption cross-sectional area, *σ*_s_, which is approximately one order of magnitude smaller than that of the visible transitions [29, 30]. Still, a perfect extinction can be achieved with structures that ensure a sufficiently long optical path-length, such as a light-trapping waveguide [31] and bulk cavity structures [32]. However, these structures present a significant limitation to applications that require efficient use of a compact sensor volume. Resonant nanostructures can mimic the similar effect without making the path-length physically long and sacrificing footprint. The following section discusses different categories of resonant structures and their design criteria to maximize the readout fidelity.

## 4 Resonant structures

### 4.1 Cavities

When an ensemble of NVs is coupled to a resonant structure, their rate of transition is enhanced by a factor given as follows:

(2) $$\frac{\Gamma }{\Gamma_{0}}=\frac{\frac{1}{2}\epsilon |E\left(\vec{r}\right){|}^{2}}{{\left(\frac{1}{2}\epsilon |E\left(\vec{r}\right){|}^{2}\right)}_{\max}}\frac{Q}{V_{\text{eff}}/{\left(\frac{\lambda_{0}}{n_{\text{diamond}}}\right)}^{3}}$$

where $\frac{\frac{1}{2}\epsilon |E\left(\vec{r}\right){|}^{2}}{{\left(\frac{1}{2}\epsilon |E\left(\vec{r}\right){|}^{2}\right)}_{\max}}$ accounts for the spatial variation of an optical field, *Q* and *V*_eff_ are the quality factor and effective mode volume of the cavity, and *λ*_0_ = 1042 nm. In the expression above, for simplicity, we assume spherically uniformly oriented quantum emitters (although there are four possible dipole orientations perpendicular to the NV axes) and take an ensemble average of the absorption rate. For a given sensing volume, the total absorption signal is enhanced by the discrete summation over all NVs, $\sum_{i}^{N_{\text{NV}}}\frac{\Gamma_{i}}{\Gamma_{0}}$ , where *N*_NV_ is the total number of NVs. When NVs are located in which the electric field energy density is concentrated and relatively uniform, the summation can be approximated as $\approx n_{\text{NV}}Q$ , where $n_{\text{NV}}=N_{\text{NV}}/V$ and *V* is the sensing volume. The readout fidelity and sensitivity improve as $\sqrt{n_{\text{NV}}Q}$ . *n*_NV_ is, more accurately, the net population of the singlet ground state, which is responsible for the total IR absorption signal, and thus is a function of optical and MW excitaitons. This figure of merit (FOM) suggests that dielectric cavities that are characterized by high *Q* would be more suitable. However, since dielectric cavities often have diffraction-limited mode volumes, plasmonic cavities with deep subwavelength-scale mode volume could be more favorable for sensing and imaging with a sub-diffraction-limit spatial resolution [26, 42]. Typically, with plasmonic structures, quenching contributes to significant loss and is detrimental for fluorescence collection. However, the quenching effect works favorably for the absorption readout, as it resets electrons back down to ^1^*E* more quickly, freeing up more electrons for absorbing subsequent IR photons. Furthermore, with plasmonic cavities, one can resolve linear dimensions close to those of nanodiamond or scanning probe. Cavity arrays provide an advantage over nanodiamond or scanning probes: the possibility of massive parallel detection scheme. Material losses and resultant low *Q* are the major limitations of plasmonic cavities. However, they may be circumvented by a recently developed self-similar dielectric cavity design with a sub-diffraction mode volume and a high *Q* only limited by radiation loss [43].

### 4.2 Metasurfaces

A second category of resonant devices are metasurfaces decorated with specifically arranged sub-wavelength structures. Metasurfaces can be understood as a surface consisting of closely spaced resonators that are coupled, giving collective phenomena that are not found in bulk materials. Such nanometer-scale-thick sub-wavelength structures create abrupt changes in electromagnetic boundaries and give rise to changes in the phase and amplitude of light at the interface. Metasurfaces are designed to in- and out-couple with a radiative field, making interrogation of NV spins with external light optimal.

**Figure 2:**
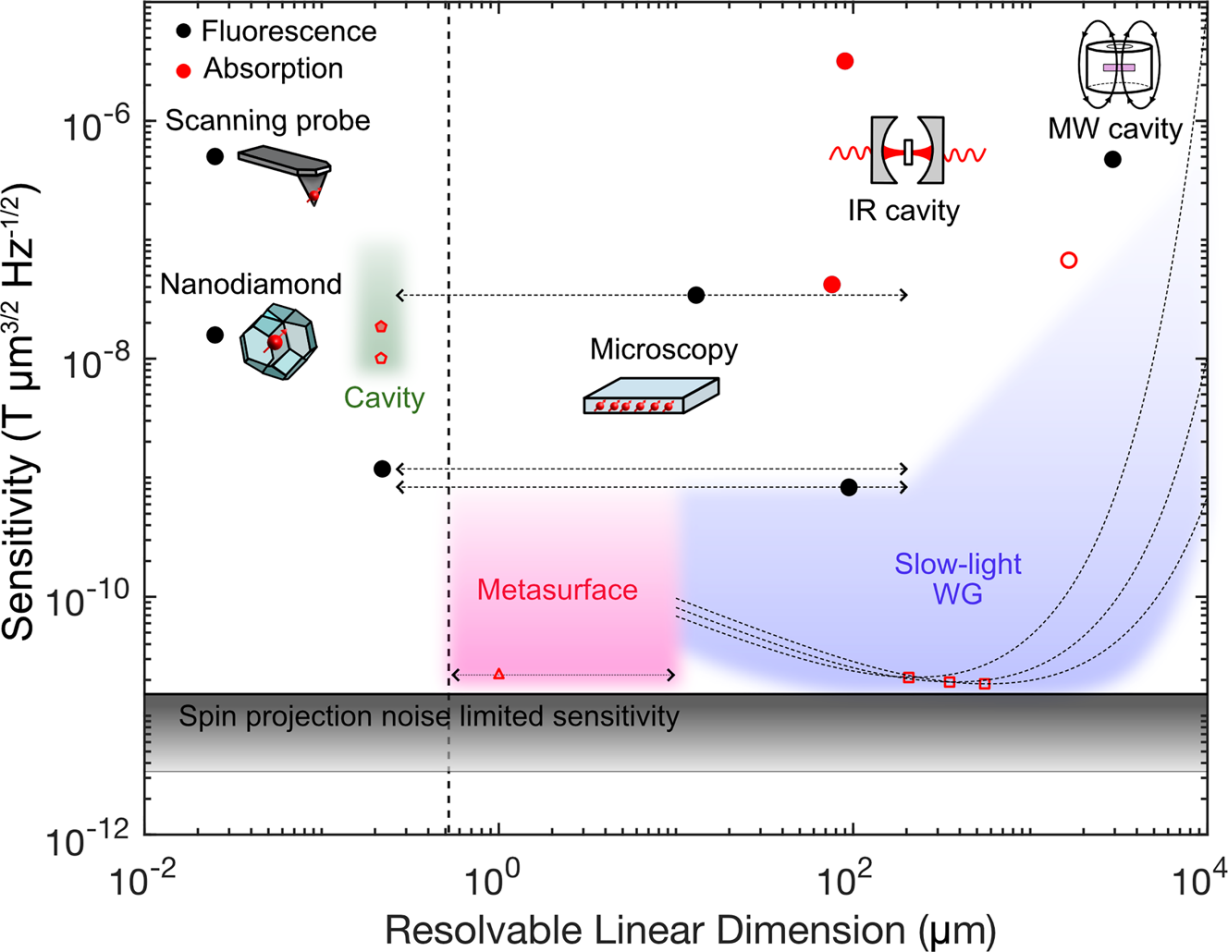
Volume-normalized sensitivities of previously explored NV magnetometers – both experimentally (solid circle) and theoretically (hollow circle) – as a function of their resolvable linear dimensions: nanodiamond [33], diamond scanning probe [34], diamond quantum microscopy [10, 12, 35], IR bulk cavities [30, 32], light trapping waveguide for IR absorption [31], and MW cavity [36]. The vertical dotted line indicates the diffraction limit of the probe beam at 1042 nm. The top and bottom horizontal black solid lines indicate the spin projection noise limited sensitivities of NVs, assuming 1-ppm concentration and *T*_2_ of 1 μs and 20 μs, respectively. Resolvable lateral spatial resolution of fluorescence-based diamond spin microscopy can range from the diffraction limit to the limit where a given sensing volume can be excited homogeneously with reasonable optical and MW powers (this range is indicated by the dotted horizontal lines). The shaded regions represent the projected sensitivities along with corresponding resolvable linear dimensions for nanophotonic quantum sensing devices that are designed for IR absorption readout (green for cavities, red for metasurfaces, and blue for slow-light WGs). Representative values for quality factors for diamond cavity structures are taken from [37–39] (pentagon). A theoretical study on a quantum sensing plasmonic metasurface that is designed specifically for a micron-scale sample predicts sensitivity near the spin projection noise limited sensitivity [40] (triangle). Representative slow-light factors and corresponding losses of dielectric WGs are taken from [41] (square). When the average number of photons interacting with an NV for a given readout time is conservatively assumed to be near 10, the projected sensitivities of slow-light WG-assisted sensors are shown in dotted curved lines for assumed slow-light factors of approximately 27, 37, and 50 [41].

The readout fidelity of metasurface-assisted sensing scales with the difference in NV absorption at 1042 nm when NVs are in *m*_s_ = |±1⟩ and *m*_s_ = |0⟩ [40], assuming the intrinsic metasurface response, |*α*_0_|^2^, is much higher than the NV absorption, |*α*_NV_|^2^:

(3) $$1/\sigma_{\text{R}}\propto |\alpha_{\text{NV}}\left(\Omega_{\text{R}}\right)|-|\alpha_{\text{NV}}\left(0\right)|$$

where |*α*_0_|^2^ is the intrinsic reflection or transmission of a metasurface without NV contribution, and |*α*_NV_(0/Ω_R_)|^2^ is the NV absorption without/with MW excitation. For a given pixel area of an imaging surface, the absorption signal improves by $\sum_{i}^{N_{\text{NV}}}|E_{i}\left(\vec{r}\right)/E_{0}{|}^{2}$ , where |*E*/*E*_0_| represents the optical field enhancement factor over the single-pass case without a resonant enhancement. Assuming a uniform concentration and distribution of NVs in the sensing volume, this value can be approximated as $N_{\text{NV}}\left\langle |\frac{E_{i}\left(\vec{r}\right)}{E_{0}}{|}^{2}\right\rangle$ , where $\left\langle |\frac{E_{i}\left(\vec{r}\right)}{E_{0}}{|}^{2}\right\rangle$ is the spatially averaged electric field intensity enhancement factor [40]. Therefore, the readout fidelity will improve as $\sqrt{N_{\text{NV}}\left\langle |\frac{E_{i}\left(\vec{r}\right)}{E_{0}}{|}^{2}\right\rangle }$ . With sensing surfaces, it is easy to scale up or down the sensing volume. The upper limit is capped by power budgets and achievable spatial homogeneity of optical and MW deliveries. The lower limit on the resolvable dimension is bounded by the diffraction limit, although additional structures can be introduced to isolate modes along the in-plane direction to achieve a lateral spatial resolution beyond the diffraction limit (thus, the dotted horizontal line for metasurfaces in Figure 2 can extend beyond the diffraction limit). In practice, it is optimal when the NV thickness is approximately the same as the thickness of a sample being probed, *t*_sample_, because increasing the NV thickness beyond *t*_sample_ increases signal only marginally while adding to the shot noise. Thus, depending on samples of interest, it is possible to engineer the photonic structure for vertically extending mode by reducing the decaying constant [40].

### 4.3 Slow-light waveguides

Slow-light waveguide (WG) structures produce a photonic bandgap, resulting in a small group velocity near the band edge. Reducing the group velocity of light increases the interaction time between the dipole and the emitted field and Purcell-enhances the rate of transition. Waveguide structures are beneficial for applications that require minimal exposure of samples to probing optical fields. The measurement contrast is given by $C=\frac{P_{\text{out}}\left(0\right)-P_{\text{out}}\left(\Omega_{\text{R}}\right)}{P_{\text{out}}\left(0\right)}$ , where *P*_out_(Ω_R_/0) is the output power collected at the end of a WG with/without MW excitation. The input light traveling in a WG is attenuated by both the intrinsic loss of the WG and NV absorption: $\frac{{\text{P}}_{\text{out}}}{{\text{P}}_{\text{0}}}={\text{e}}^{-2L\left(\alpha_{\text{WG}}+\alpha_{\text{NV}}\left(\Omega_{\text{R}}\right)\right)}$ , where *L* is the length of a WG, *α*_WG_ is the intrinsic attenuation coefficient of a WG, and *α*_NV_ is the attenuation coefficient associated with NV absorption of 1042 nm light (∝*σ*_*s*_*n*_NV_). Thus, when it is assumed that $\frac{P_{\text{out}}\left(0\right)}{P_{0}}\approx {\text{e}}^{-2L\alpha_{\text{WG}}}$ (i.e., WG attenuation dominates NV absorption without MW excitation), $C\approx 1-{\text{e}}^{-2L\alpha_{\text{NV}}(\Omega_{R})}\;$ . The spin contrast improves with increasing *α*_NV_, and with the slow-light effect, *α*_NV_ is modified as follows.

(4) $$\alpha_{\text{NV}}\to \alpha_{\text{NV}}\frac{\epsilon |E{|}^{2}}{{\left(\epsilon |E{|}^{2}\right)}_{\text{max}}}\frac{\left(c/n\right)}{v_{\text{g}}}\frac{A}{A_{\text{eff}}}$$

where *ϵ* is the dielectric constant of diamond, |*E*|^2^ is the electric-field intensity at the position of an NV sensor, *v*_g_ is the group velocity, *A* is the fractional cross-sectional area of a WG occupied by NVs, and *A*_eff_ is the effective mode area of a slow-light WG. To maximize the spin contrast, one needs to increase the interaction time that an individual NV sensor has with an optical field by lowering the group velocity of probe light. Furthermore, it is desirable that an electric field intensity over a sensing volume is nearly uniform, and the filling factor of NVs is as close as to the volume occupied by the optical field. The relevant FOM for maximizing the readout fidelity is given by $C^{2}n_{\text{avg}}\propto e^{-2L\alpha_{\text{WG}}}{\left(1-{\text{e}}^{-2L\alpha_{\text{NV}}}\right)}^{2}$ .

Unlike a sensing surface, a WG-based sensor cannot be scaled up or down by adjusting its length while maintaining the volume-normalized sensitivity. There exists a minimum WG length necessary to generate sufficient spin-dependent extinction. On the other hand, it is not ideal to make the WG infinitely long as the signal collected at the end of the WG diminishes exponentially due to its loss. Therefore, there exists an optimal WG length that can gives optimal readout fidelity. Thus, in Figure 2, the predicted slow-light WG-assisted volume normalized sensitivity has a nonlinear trend with an optimized sensitivity point. A greater slow-light factor comes with a larger loss. For applications that do not require fine spatial resolution, minimizing loss is important; on the other hand, for applications that require small spatial resolution, optimizing the slow-light factor is preferred.

## 5 Discussion

When spin states are read-out via absorption, resonant nanophotonic engineering can assist in reaching near-unity fidelity and near-spin-projection-noise-limited sensitivity. Nanophotonics has a particular advantage for micro- and nanoscale sensors where efficient use of a sensor volume is key, as shown in Figure 2. These applications preclude scaling in NV number and instead require high fidelities from spins within confined volume. As indicated by the shaded regions in Figure 2, the range of resolvable linear dimensions of resonant devices may span from the below-diffraction scales to diamond chip lengthscales.

As atom-sized quantum sensors, NVs measure their environment with a spatial resolution beyond the diffraction limit, but resolving this information requires methods beyond direct optical imaging. The spatial resolution of the optical readout is limited by diffraction, which gives the minimum distance required for two features (e.g. emitting dipoles) to be separately identified in the far field. Resonant nanophotonic structures can be utilized to achieve sensing beyond the diffraction limit. One avenue is to encode spatial location in the spin degree of freedom, for example, with spatially varying static magnetic field [44, 45], or with resonant control fields that are confined at nanometer scales [46]. Metasurface layers can couple to neighboring electrodes, or plasmonic structures may double as electrodes. Such electrodes can be used to launch various waves to control spins. NV spins are found to be susceptible to other fields that can be better spatially confined such as acoustic waves and electric fields [47–49]. Such methods can achieve sensing and imaging resolution below diffraction-limited scales. Finally, nanophtonic quantum sensing is compatible and can be combined with techniques such as STED [50] and RESOLFT [51] that control the excitation field on sub-diffraction scales. In the other direction, the enhanced spin-optic coupling can extend the upper limit of resolvable linear dimension as well. The resonant structures reduce the power consumption necessary to drive the system near its saturation level. This is beneficial for applications where heat mitigation is critical under optical pumping.

An important advantage to note about nanophotonic-enhanced absorption readout is the spin-dependent optical response that can be easily engineered and manipulated. Spin-dependent signals can be manifested in the form of all three properties of light: polarization, phase, and amplitude. Polarization of an optical field can be utilized to interrogate a specific NV orientation to reduce the background signal. Under a coherent optical field excitation, spin-dependent phase and amplitude changes of the optical signal may be compatible with compressive sensing and computational imaging techniques to gain multidimensional information.

For successful implementation of resonant nanophotonic structures, there are challenges that need to be considered. First, spatially inhomogeneous electric field profiles created by resonant structures may produce non-negligible spatial variability in NV dynamics and non-uniform sensitivity over space. When NVs are incorporated into nanostructures, position-dependent coherence times of NVs may also contribute to spatially varying sensitivities. One way to mitigate this challenge is to engineer the position of NVs in diamond with 10 nm–scale spatial precision [52–54] via growth with delta-doping [55], focused ion implantation [56], and high-energy implantation with high aspect-ratio masks [54, 57]. Second, nanofabrication processes modify the surface properties of diamond, potentially resulting in deleterious effects on near-surface NV centers. These include the production of electric and magnetic noise sources that can affect NV optical coherence, charge stability, and spin coherence. The compromised optical coherence is not an issue for sensing applications as most protocols do not rely on coherent emission, and THz-scale phonon broadening dominates at room temperature generally. NV spins are vulnerable to various sources of spin decoherence, including unconverted nitrogens (P1 centers), surface trapped charges, and vacancy complexes with unpaired electrons that form fluctuating spin baths [58–62]. Interfaces and surface termination are reported to affect the charge stability of NV centers, which has been addressed via both passive and active methods [63–65]. The need for a better understanding of surface and interfacial effects and the optimization of diamond growth and NV production warrant further studies.

## 6 Conclusions

NV centers are room-temperature quantum systems with exceptionally long coherence times, which eliminate the need for vacuum or cryogenic systems for sensing. As a result, NV solid-state spin sensors can be brought in close proximity to samples and make the detection of magnetic fields at the quantum level under ambient conditions possible. Achieving near-unity optical readout fidelity for room-temperature ensemble sensing has remained elusive, plagued by fundamental and technical challenges. Combining the resonantly enhanced spin-optic coupling with absorption readout, one can expect to bridge the gap between what has been achieved with present methods – nanodiamonds, scanning probes, bare diamond plates, and bulk cavity structures – and the sensitivity limit only bounded by quantum noises.
